# Visual Sensing for Urban Flood Monitoring

**DOI:** 10.3390/s150820006

**Published:** 2015-08-14

**Authors:** Shi-Wei Lo, Jyh-Horng Wu, Fang-Pang Lin, Ching-Han Hsu

**Affiliations:** 1 National Center for High-Performance Computing, No. 7, R&D 6th Rd., Hsinchu Science Park, Hsinchu City 30076, Taiwan; E-Mails: LSW@nchc.narl.org.tw (S.-W.L.); jhwu@nchc.narl.org.tw (J.-H.W.); 2 Department of Biomedical Engineering and Environmental Sciences, National Tsing Hua University, No. 101, Section 2, Kuang-Fu Road, Hsinchu 30013, Taiwan

**Keywords:** visual sensing, urban flood monitoring, water level fluctuation

## Abstract

With the increasing climatic extremes, the frequency and severity of urban flood events have intensified worldwide. In this study, image-based automated monitoring of flood formation and analyses of water level fluctuation were proposed as value-added intelligent sensing applications to turn a passive monitoring camera into a visual sensor. Combined with the proposed visual sensing method, traditional hydrological monitoring cameras have the ability to sense and analyze the local situation of flood events. This can solve the current problem that image-based flood monitoring heavily relies on continuous manned monitoring. Conventional sensing networks can only offer one-dimensional physical parameters measured by gauge sensors, whereas visual sensors can acquire dynamic image information of monitored sites and provide disaster prevention agencies with actual field information for decision-making to relieve flood hazards. The visual sensing method established in this study provides spatiotemporal information that can be used for automated remote analysis for monitoring urban floods. This paper focuses on the determination of flood formation based on image-processing techniques. The experimental results suggest that the visual sensing approach may be a reliable way for determining the water fluctuation and measuring its elevation and flood intrusion with respect to real-world coordinates. The performance of the proposed method has been confirmed; it has the capability to monitor and analyze the flood status, and therefore, it can serve as an active flood warning system.

## 1. Introduction

### 1.1. Urban Floods

Climate extremes and changing weather, which are associated with global warming and climate change, have led to a significant increase in the frequency and severity of floods [1]. Meteorological observations have shown that in many cities worldwide, precipitation has become heavier and more variable [2]. The overall incidence of extreme rainfall has also increased gradually, and this has caused heavy losses to human life and property in flooded areas [3]. Owing to their increasing incidence and severity, floods are expected to be a continuous threat in most cities worldwide [4,5]. Nowadays, flood warning systems are being widely used for monitoring and forecasting flood disasters and water resources. However, one-dimensional measurement data of the water level and streamflow from gauge stations are insufficient for representing actual runoff-land interactions. Moreover, existing flood risk models have low specificity and are insufficient for the analysis of small districts. Furthermore, decision-makers cannot obtain sufficient visual field information for disaster control and hazard reduction. Currently, owing to large-scale urbanization, ~50% of the world’s population lives in crowded cities [6]. However, the concentrated populations and economic activities of urban areas may also make them more vulnerable to the impact of floods on account of increased difficulties in evacuation and sheltering procedures. Thus, to assist decision-makers in analyzing a situation for public disaster warning or hazard reduction actions, the real-time visual monitoring of urban flood events is a very important tool. In this paper, based on the needs of decision-makers for urban flood control, we propose the use of a visual sensing technique based on network cameras for hydrological management to realize intelligent surveillance and warning of river overflows. As a sensing system for urban intelligent flood control and disaster reduction, this tool provides flood warnings and real-time information to disaster relief sectors for disaster reduction actions.

### 1.2. Flood Monitoring

#### 1.2.1. Gauge Sensing

An urban flood hazard occurs because the surface runoff caused by heavy rainfall cannot be relieved in a timely manner. This hazard involves various aspects, including structural erosion; building damage; water pollution; interruption of social and economic activities, transportation systems, and communication networks; and loss of life and property. Climate change includes extreme weather patterns, leading to an increased frequency of flood hazards. Among all natural disasters, flood hazards are the most serious in terms of the number of people impacted and the deaths caused [7,8]. Therefore, studies on flood hazards have attracted significant attention in some scientific fields, such as those focusing on water resources and natural disasters. Currently, dynamic flood monitoring has been extensively used in flood warning systems. Such systems mainly obtain data from a number of gauge stations, such as water level measurement stations, precipitation stations, and meteorological radar stations in catchment areas [9,10,11,12,13]. The data are mainly those of water-level fluctuation and the amount and precipitation distribution. In addition to their use in real-time flood monitoring, these data can also be applied to forecast future water-level fluctuations [14,15]. The flood warning system monitors hydrological variables as well as their time derivatives to provide disaster prevention sectors with advance flood information for flood management and relevant disaster reduction actions. In this way, the flood impact can be mitigated as much as possible.

When a flood event occurs, the simplified water level, precipitation data, and observational evidence as well as streamflow models are available for disaster prevention sectors to determine disaster reduction actions accordingly. However, the water level data contains only one spatial dimension, a point-based view of the water surface, that cannot accurately represent the actual runoff-land interactions such as the spatial dynamics of the surface water extent. Moreover, whether monitored precipitation would cause a regional flood mainly depends on whether the regional runoff water can be discharged in a timely manner. For example, in an urban environment, the surface runoff water is collectively discharged to the sewer or a neighboring river. Thus, short heavy rain might be enough to cause a rapid raise in a small river’s water level and lead to overflow or even a flood disaster [16]. Therefore, the real-time field images of water levels, bank overflows, and the features of the surrounding ground surface and buildings in the runoff, in combination with data from precipitation and water level records, will be more helpful to disaster-relieving operations.

#### 1.2.2. Remote Sensing

In addition to data collection from gauge stations, remote sensing technologies such as optical imagery and radar imagery are also widely used for measuring the wide-area water level and scope for defining a flooded area [17,18,19,20,21]. Remote altimetry technology can be used to continuously measure the water level variation within a large area and can thus be used to extensively monitor an entire flood event [22,23,24]. Another advantage of this technology is that it remotely acquires data of absolute water elevations, which is helpful for the integration of flood management and for environmental science research. Comparatively speaking, gauge-based ground monitoring is limited by station distribution and numbers, which may result in uneven coverage of a floodplain. Moreover, the measured water level is a relative level, and it needs to be standardized before comprehensive data pooling among different stations can be performed. Currently, remote sensing images of water levels show decimeter-level measurement accuracy, and the transmission is almost real-time in nature [13,18]. However, thus far, studies on the remote measurement of water levels have mainly focused on large-area waterbodies, such as oceans, interior lakes, lagoons, and large rivers (width > 100 m). Only a few studies have focused on small-scale river branches (width < 40 m) in urban areas, which typically zigzag through the urban areas and can be easily blocked by buildings [25,26]. Flood detection in urban areas is greatly improved using remote sensing techniques. Recent advances in Earth Observation (EO) are improving the capability of detecting flooded zones in urban areas via high-resolution synthetic aperture radar (SAR) [19,27,28,29,30]. Additionally, a digital elevation model (DEM) created using an X-band sensor (e.g., TerraSAR-X and COSMO-Skymed Constellation) is widely used for flood prediction and monitoring. A satellite can transmit short-wavelength microwaves to acquire data throughout the day regardless of the weather conditions [31,32]. This, along with the increasing computational power, is facilitating the real-time integration of SAR-derived water levels with operational flood forecast models via data assimilation (DA) techniques [33,34,35,36,37,38]. The sensor observations are integrated with the model by DA techniques to obtain a 3D description of the flood for a forecast as well as for the flood warning systems. Furthermore, the remotely measured water level data still need to be verified to ensure accuracy using ground measurement data [39]. Owing to the restriction of orbital cycles and inter-track spacing of satellite movements, the limitation of remote measurement data in continuous monitoring and the observation of fixed points is another key problem. Thus, it is difficult to use remote sensing technology for long-term and near-real-time water level measurement at fixed points in small rivers in urban areas. Therefore, regardless of its real-time monitoring capability and high accuracy, remote sensing technology is not well-suited for urban flood prediction in terms of cost and timeliness.

#### 1.2.3. Flood Risk Mapping

Model analysis has been widely applied in studies on flood risk. In this type of analysis, hydrological data such as water level and precipitation from monitoring stations is combined with remote measurement images and geographic models to analyze and simulate flood events. In this way, this analysis derives risk information from a probabilistic viewpoint, which can be assessed with regard to various scenarios such as potential flood depth and coverage within a certain geographic area, and it provides flood risk assessment and risk mapping to identify zones that are vulnerable to flooding in advance [16,40,41,42,43]. For flood management and policy implementation, the advance acquisition of flood risk assessment information, such as the flooding probability, degree, location, and height, enables the government to implement disaster management practices in advance. Furthermore, results from the flood model analysis can be used for disaster reduction. For example, for urban flood relief, these results can be considered and used for the determination of settings and operational regimens of flood discharge valves and pump stations [44,45]. On account of the possible geomorphological alteration caused by changes to buildings or streets, or the construction of new ones, the model must be periodically updated for urban flood risk mapping. Nevertheless, risk mapping is used for large-scale risk assessment, which is not specific or conducted in real time for urban local-scale assessment. In addition, it is difficult to verify the analysis results with the actual runoff status of the streets. Thus, this method remains insufficient for enabling decision-makers to conduct immediate and accurate disaster reduction actions.

### 1.3. Proposed Flood Visual Sensing

*In situ* gauge measurement is the most basic and extensively used means for obtaining hydrological information. Although this measurement can quantitate flow dynamics, such as river flood discharge or overflow, it lacks dynamic information regarding the actual spatial change in runoff, such as floodplain flow and bank overflow. In most places where conditions are not suitable or funds are lacking to build a gauge station, CCTV monitoring is used as an alternative solution. Furthermore, because it is difficult for decision-makers to clearly understand the field flood situation based on these measurement data, the decisions made might inevitably be unsuitable in the actual situation. Although the use of remote sensing images can overcome the in situ gauge measurement’s disadvantage of incomprehensive flood monitoring and can reach decimeter-level accuracy for water level measurement, it cannot realize long-term continual monitoring of urban small-scale flood events and accurately measure the water level changes in small rivers.

Closed-circuit television (CCTV) has been widely used in river monitoring [46], water level measurement [47], and flood modeling and flood emergency management [48], which employ remote video surveillance images to obtain dynamic field information of streamflow or continuously filmed images to analyze the surface stream velocity of water [49,50]. The detection of the water body using seed-guided graph-based image segmentation and the details of the algorithm are described in Section 2.2. The image segmentation technique is currently widely used in various fields of science and engineering. It can roughly be divided into boundary-based, graph-based, and statistical-based methods [51,52,53,54,55]. The goal of an image segmentation technique is that it groups pixels as multiple segments that represent the visual perception meaning from an image. The pixels within a segment have similar properties such as color and texture. This is typically used to identify objects or other relevant information in digital images.

**Figure 1:**
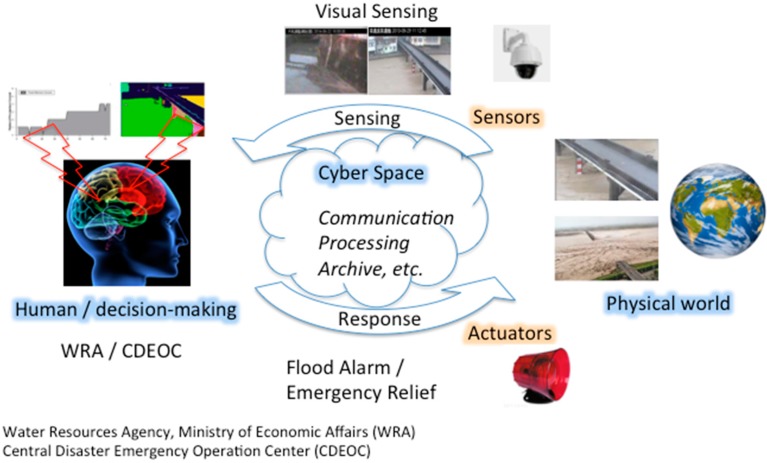
Flowchart of visual sensing for flood events. The system utilizes visual sensor technology to automatically analyze the monitoring image to obtain information regarding the field water level and runoff region. According to the analysis results of high-risk areas, the system automatically alerts the ultimate decision-makers, who will then choose suitable disaster reduction actions based on the geographic images and runoff data.

Therefore, real-time images provide more useful dynamic field information to support improved decisions from disaster management agencies in optimizing their emergency responses. In this paper, we propose a visual sensing application for real-time image-based monitoring of flood fluctuations in small urban riverine areas. The sensed data, including visual data, are used for supporting decision-making for disaster reduction, as shown in Figure 1. A camera can obtain real-time field images of runoffs and automatically analyze the field water level situation. Therefore, the proposed visual sensing application can be used for flood detection and provide data regarding water level changes, which will help disaster management agencies to rapidly and accurately understand the flood situation and initiate disaster reduction actions accordingly.

## 2. Intelligent Urban Flood Visual Sensing of Smart Cities

In a modernized smart city, information and communication technology (ICT) has been widely applied in areas such as transportation management, communication, resource management, and instrument information. Technologies such as cyber-surveillance and sensor networks are also commonly employed in a variety of intelligent applications [9,56,57,58]. In addition to long-term water management, the hydrological management of a smart city includes key components, such as short-term emergency flood alarm and disaster reduction activities, which are decided by the departments of disaster prevention and hydrological management. One of the most important steps in the flood alarm system is the process of providing accurate and concise field information to the decision-makers through ICT. For water resource management and maintenance, monitoring systems have been used in important river zones to enhance the surveillance initially performed by humans. This provides continual monitoring while greatly reducing human resource consumption. However, all real-time monitoring images currently still require a one-by-one manned check to determine the occurrence of river overflow after being sent to the central system. Therefore, this system does not function for intelligent early warnings.

This study aims to address the question of how to utilize a visual sensing system to assist in early warnings for urban floods. Figure 1 shows a flowchart of the application of intelligent visual sensing in an urban flood management system. First, real-time field images are automatically analyzed to determine the flood severity by image processing with set virtual markers; the data are then sent to decision-makers, along with the field images and water level results for reference, to help in accurately and rapidly providing warnings and responses.

The main software modules of the flood visual sensing system are as follows: Section 2.1 an event-based triggered visual sensing system for determining image quality and verifying the existence of a waterbody; Section 2.2 detection of the runoff for determining the waterbody region using an image segmentation method; and Section 2.3 calculation of runoff fluctuation for analyzing the flood risk degree using waterbody region data based on a preset virtual marker or the scales of *in situ* water-level rulers.

### 2.1. Flood Visual Sensing

**Figure 2:**
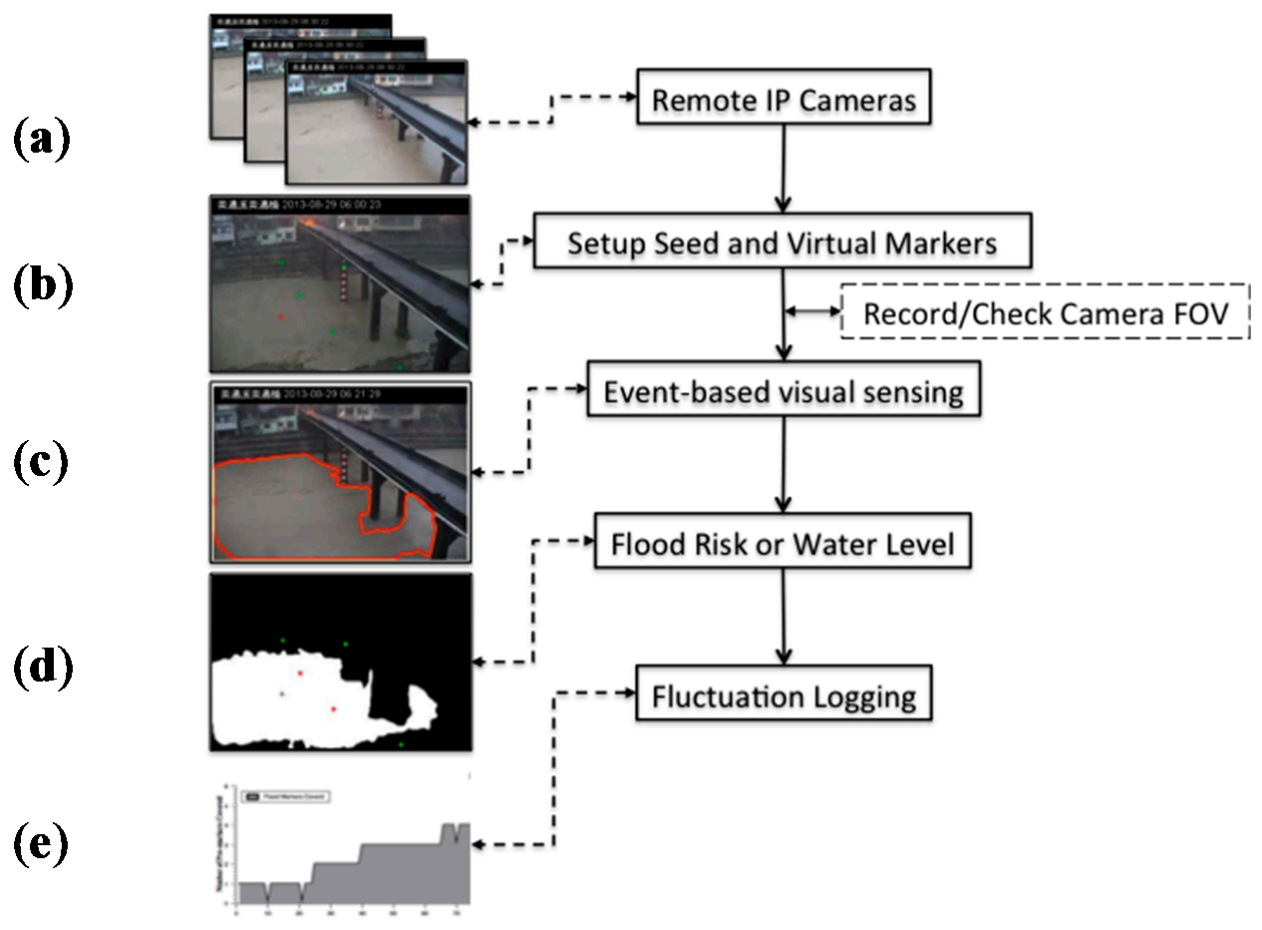
Flowchart of visual sensing process for floods. Before image analysis, (**a**) check whether the camera’s field of view (FOV) is correct; (**b**) set up a virtual seed and markers; (**c**) determine whether to carry out waterbody detection using an event-based trigger; and (**d**) evaluate the water level fluctuation tendency; (**e**) Water level variation based on the computation of waterbody detection data combined with virtual markers.

A flowchart of the visual sensing used in this study is shown in Figure 2. All of the *in situ* remote monitoring cameras were situated at distances of at least tens to hundreds of meters from the watercourse. Most of them were located at a higher place behind the riverbank to improve their survival rate during disasters while ensuring that the FOV of the cameras could cover the entire monitored scene. Figure 2a,b show the input monitoring image and the image with preset virtual markers from an actual operation, respectively. The red “o” and green “o” represent the locations of the seed and virtual markers, respectively. The red outline in Figure 2c indicates the detected region of the waterbody, whereas Figure 2d shows the waterbody contour expressed as a binary image after background removal. In the binary image (Figure 2d), the virtual markers covered by the waterbody are denoted with a red “*”; the seed location is marked by a black “+”. Figure 2e shows the post-analysis water level fluctuation data.

The monitoring images of each site were compressed in JPEG format with a resolution of 352 × 288 (CIF). These images were returned to the central management center at a frequency of one image per minute, as shown in Figure 2a. Before the initial visual sensing process, each camera station was set with a virtual seed and several virtual markers in advance, as shown in Figure 2b. The seed is used to analyze the waterbody surface texture and guide image segmentation of waterbodies, whereas virtual markers are used to indicate the boundary of an actual space (for example, marking over the length of a water-level ruler on the bridge pier or the surface of a riverbank). The flood risk of the runoff can be evaluated according to the number, proportion, and sequence of virtual markers covered by the waterbody. Meanwhile, the FOV parameters of the cameras can also be recorded and checked during the visual sensing work. If the parameters do not meet the requirement, the cameras should be automatically reset to the default FOV parameters to ensure the FOV consistency of images and virtual markers throughout the visual sensing process. The FOV parameters, which can be automatically retrieved from the cameras, consist of the pan angle, tilt angle, and zoom ratio (pan/tilt/zoom, PTZ). The record/check FOV procedure in the overall workflow is shown in Figure 2.

The visual sensing triggered by event-based rules (rules in Table 1 and sensing result in Figure 2c) is used to determine whether input monitoring images are suitable for visual detection. If the requirement is met, the images are then segmented to separate the waterbody region from the image. However, the cameras in the visual sensor network (VSN) might have different specifications, functions, and configurations (e.g., with/without defog, nighttime infrared (IR) imaging, infrared auxiliary radiation source, and nighttime auxiliary lighting). Moreover, the quality of captured images is subject to the impact of weather (e.g., insufficient daylight, heavy fog caused by atmospheric suspended particulates and water vapor). Therefore, the designing principle of event-based trigger rules is based on whether the screened image content is analyzable. For example, first, monitoring images with excessively low image contrast (likely caused by insufficient brightness, non-IR thermal images captured at nighttime, or blurred images caused by heavy fog or rain) or low brightness (nighttime or insufficient daylight) can be excluded because they are non-analyzable. Subsequently, the texture analysis of the waterbody surface in the image is performed to verify the existence of the waterbody. Finally, waterbody region segmentation (Figure 2d) and flood severity analysis (Figure 2e) are conducted.

**Figure 3:**
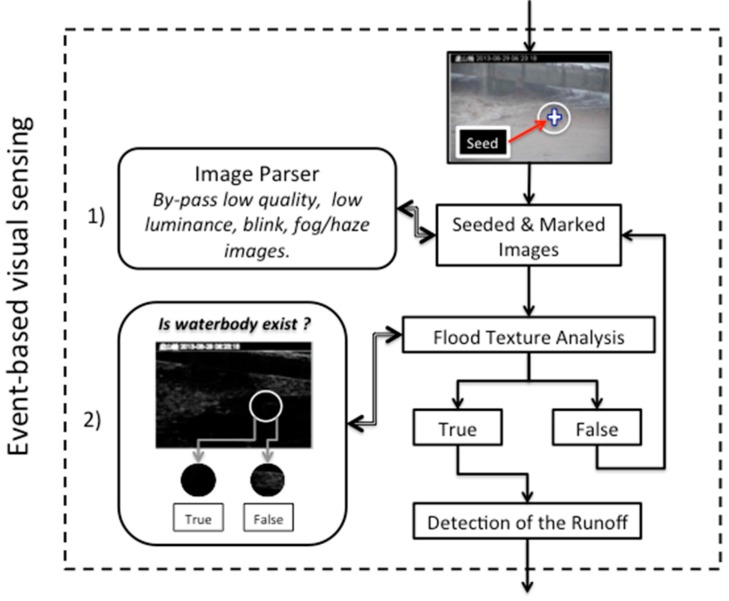
Flowchart of sensing triggered by using event-based rules. All remote monitoring images are subject to a two-stage screening: (1) quality analysis of images for screening of images that cannot be analyzed, and (2) surface texture analysis of the seeding region of interest (ROI) to verify the existence of a waterbody. Only images that are subject to the two-stage screening are used for the subsequent waterbody region detection.

The event-based trigger mechanism minimizes the discrepancies among the images to be analyzed, which are caused by the use of different monitoring devices and environmental conditions. This mechanism makes simultaneous or individual control possible for the flood risk assessment of each individual monitoring station, thereby reducing the use of computing resources. The procedure of the event-based trigger mechanism is shown in Figure 3. It is described in detail in Event-Based Trigger.

#### Event-Based Trigger

When visual sensing is used in outdoor conditions that rapidly change, the image-capturing device and imaging quality determine the success of the subsequent analysis. However, most hydrological monitoring stations do not use advanced intelligent cameras or infrared thermal cameras with night vision and fog penetration features. Therefore, the unanalyzable images (or poor quality images) might be transmitted to the central warning system. Poor quality images are defined based on “analyzability” and “identifiability.” Because various factors are associated with the poor quality of unanalyzable images, it is difficult to design and implement algorithms for image analysis in terms of their sources. As a result, in this study, only images that could be used for visual analysis (images with significant perceptual identifiability) were considered analyzable. Accordingly, a universal monitoring image parser was designed based on this standard. In general, poor image quality is caused by insufficient luminance (e.g., images captured at night without night vision), excessive luminance (e.g., direct sunlight, strong light reflected by glass walls of buildings, interference of high-beam lights of vehicles, *etc.*), insufficient contrast (images obscured by thick fog, or images with a background that is too bright or too dark), hardware or network defects (blank images due to a signal-capturing interruption; these are usually a black, green, or blue screen), a dirty camera lens (contaminated mirror surface, spilling of rain water, *etc.*), and so on.

Unanalyzable images can be attributed to various factors. Therefore, it is not possible to design algorithms specific to every cause. Instead, an outcome-based approach can be applied, namely, a parser is used to reserve analyzable images and remove poor-quality ones. In this study, analyzable images are defined as images with luminance and contrast within a certain visible range; the content in these images has high visual identifiability on account of the appropriate luminance and contrast. The image property parameters used in the image analysis are shown in Table 1. The range of each parameter is obtained from the visual-detecting experience of the users. The work process of the parser is as follows:

*// **Image Parse for Day/Night/Low−visibility/Fog−rain−noise/Water body.//****// **Range from prior.****Limit_PercentOfDarkSamplePixels=50;  // dark % of sample pixels (pixel intensity < 50)**Limit_ImVisibility=80;         // mean intensity of image**Limit_ImVisibilityOfSamplePixels=80;  // number of sample pixels (pixel intensity < 80)**Limit_DarkChannelAvg=60;       // mean intensity of dark channel image**Limite_SeedROITexturePrc=2;      // Edge of seed ROI > 2% = riverbed or ground.**// **Image parser.****if (PercentOfDarkSamplePixels > Limit_PercentOfDarkSamplePixels)** return // **Blank image will stop here****elseif (ImVisibility < Limit_ImVisibility || ImVisibility > Limit_ImVisibility+100)** return // **Too bright or too dark image will stop here****elseif (ImVisibilityOfSamplePixels < Limit_ImVisibilityOfSamplePixels || ImVisibilityOfSamplePixels > Limit_ImVisibilityOfSamplePixels+100)** return // **Too bright or too dark of sample pixels will stop here****elseif (DarkChannelAvg < Limit_DarkChannelAvg || DarkChannelAvg > Limit_DarkChannelAvg+100)** return // **Too foggy image will stop here****elseif (SeedROITexturePrc > Limite_SeedROITexturePrc)** return // **Too much/long edge of seed ROI,riverbed or ground,will stop here****else** // **Parse Passed. Go to flood detection**.**end*

**Table 1:** Parser items and its visual meaning, applied condition, and processed example.

Parser Item	Function	Object	Image
PercentOfDarkSamplePixels	$\frac{\sum^{\text{​}}(\left(\left(Inst.\left(x,y\right)\in SamplePixels(x,y\right)\right)\leq 50)}{\sum^{\text{​}}SamplePixels\left(x,y\right)}\times \;100\%$	Blank Image, Luminance	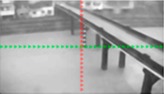
ImVisibility	$\frac{\sum_{i=0}^{N}Inst._{i}\left(x,y\right)}{N}$	Luminance, Contrast	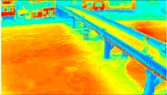
ImVisibilityOfSamplePixels	$\frac{\sum_{i=0}^{N}Inst._{i}\left(x,y\right)\in SamplePixels\left(x,y\right)}{N}$	Luminance, Contrast	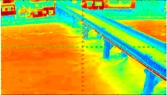
DarkChannelAvg	$\frac{\sum_{i=0}^{N}DarkChannel\_Inst._{i}\left(x,y\right)}{N}$	Fog/Haze	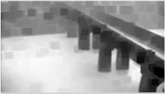
SeedROITexturePrc	$\frac{\sum_{i=0}^{N}Inst._{i}\left(x,y\right)\in EdgeOfROI}{\sum^{\text{​}}ROI\left(x,y\right)}\times 100\%$	Waterbody	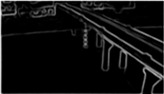

The percentage of dark sample pixels (PercentOfDarkSamplePixels) is the percentage of sampling points with intensity (here, the pixel intensity is the luminance of the HSV color space) value below 50 in the group of sample pixels. The sampling points are located on the horizontal line and perpendicular line that pass through the image center with an interval of 10 pixels. Through this simplified method to reduce the number of sampling points, the blank images and unmanageable images with insufficient luminance can be very quickly detected and filtered. ImVisibility is the average overall luminance of the image, which can be used to roughly determine whether the luminance and contrast of an individual image are suitable for visual detection. The luminance of sample pixels (ImVisibilityOfSamplePixels) is detected a second time in terms of the average luminance through sampling point detection. Even when the average luminance of an image meets the visual requirement, this may possibly be a result of averaging excessively bright or excessively dark pixels. This situation usually occurs in monitored areas where both bright reflective surfaces and shadows from shelters exist. The parameter DarkChannelAvg estimates the fog density following the dark channel prior theory [59]. The dark channel prior can obtain the distribution and density of the fog in images. Similar to the previously described method of using the average luminance to analyze the image luminance, herein, we adopt the average intensity of dark channels in an image to represent the fog density in the given image. After the first stage of image analysis, the second stage of texture analysis confirms the presence of a waterbody in images. The SeedROITexturePrc value is used to analyze the surface texture of the surrounding area, namely, the region of interest (ROI), of a seed. ROI refers to the seed-centralized image area with a radius of 25 pixels. The principles for estimating the waterbody surface are as follows. If the surface texture in a ROI area contains a significant number of geometric outlines, such as an edge and a boundary, the probability that this area is a riverbed or ground is high; otherwise, a texture that is smooth or contains a small proportion of geometric outlines indicates high probability of a waterbody. After the first screening stage, which ensures that all included images are visually analyzable, the determination of waterbody presence in the second stage can be easily simplified as the texture analysis of seed ROIs using the seed location for guidance. After the two stages of image property verification, two crucial steps—water range detection and water level analysis—can be initiated.

### 2.2. Detection of Runoff

After confirming the image quality and waterbody existence, the runoff region in the image is separated using seed-guided graph-based image segmentation. The detailed procedures used in this study are as follows. First, the image is subdivided into multiple independent segments using the graph-based segmentation method. Next, the independent segment containing the waterbody is identified under the guidance of the seed point. Because the water body texture has already been verified, the difficulty of identification and probability of false detection are significantly reduced. In addition, because the graph-based segmentation separates the image segments based on strong visually perceived characteristics, this method provides outstanding robustness for images with light attenuation and noise, even in the presence of rain and mist.

**Figure 4:**
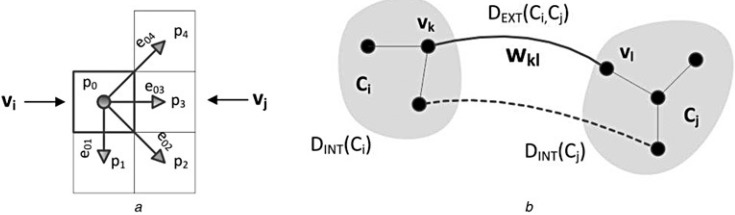
Principle of graph-based image segmentation (referred from [60]) based on an image graph and its components: (**a**) edges between the pixels and its weight, $e_{0i},\;i=1,\ldots ,4$ for any image pixel $P_{0}$; (**b**) relationships of the internal distance, $D_{INT}$, and mutual external distance, $D_{EXT}$, between two spanning tree elements, $C_{i}\;and\;C_{j}$, where $w_{kl}$ is the absolute distance of the RGB vector between the two vertices and not the Euclidean distance in space.

The solutions of graph-based image segmentation can be divided into four groups: minimal spanning tree (MST)-based methods, graph-cut methods, shortest path methods, and random walk theory based methods [60]. The graph-based segmentation algorithm used in this paper is mainly solved by using the MST-based method according to Felzenszwalb’s theory [61]. The algorithm considers an individual image as an undirected graph (G); then  G=(V,E), where $\;V=\left\{v_{k}\right\}$is the vertex collection in the graph (namely, the pixels in the images) and $\;E=\left\{e_{kl}=\left(v_{k},v_{l},w_{kl}\right)\right\}$ is the collection of vertex-connecting edges in the graph. In addition, $w_{kl}$ is the weight of adjacent vertices $v_{k}$ and $v_{l}$ on the edge, and it reflects the dissimilarity of these two adjacent vertices (see Figure 4a). According to the rule of MST, vertices in the graph can be connected as sub-tree components (C) by the edge with the smallest edge weight. $D_{INT}\left(C\right)$ denotes the internal difference and $D_{EXT}(C_{i},C_{j})$, the external distance of components; these parameters are used to decide whether adjacent components should be combined or separated (see Figure 4b).

Here, $w_{kl}$, the absolute distance of the RGB vector between the two vertices (namely, the absolute distance of the color between the two pixels and not the Euclidean distance in space) is the weighted value of the edge:

(1) $$w_{kl}=\sqrt{{\left(r_{k}-r_{l}\right)}^{２}+{\left(g_{k}-g_{l}\right)}^{２}+{\left(b_{k}-b_{l}\right)}^{２}}$$

The internal distance is defined as the maximum edge weighted value inside the component:

(2) $$D_{INT}\left(C_{i}\right)=\underset{v_{k},\;v_{l}\in C_{i},e\in MST\left(C,E\right)}{\max}w_{kl}$$

The external distance is defined as the minimum edge weighted value between all vertices of the two adjacent components, *C_i_* and *C_j_* . If the two components are not adjacent, this parameter is defined as $D_{EXT}=\infty$:

(3) $$D_{EXT}\left(C_{i},C_{j}\right)=\underset{v_{k}\in C_{i},v_{l}\in C_{j},\left(v_{k},v_{l}\right)\in E}{\min}w_{kl}$$

Thus, the pairwise comparison predicates of the two components are as follows:

(4) $$PD\left(C_{i},C_{j}\right)\{\begin{array}{l} True\;\;\;\;if\;D_{EXT}\left(C_{i},C_{j}\right)>\;\underset{\;}{\min}\left(D_{INT}\left(C_{i}\right)+\tau \left(C_{i}\right),D_{INT}\left(C_{j}\right)+\tau \left(C_{j}\right)\right)\\ \;\;False\;\;\;otherwise \end{array}$$

According to above predication (PD), if the external distances of the two components are longer than the minimum internal distance, they are predicated as independent components; otherwise, they should be combined into one single component. The threshold function τ(*C*) = *k/|C|*, where |C| is the number of elements in C. A smaller constant *k* leads to excessive segmentation that results in smaller final components; conversely, a larger constant *k* leads to segmentation that results in larger final components. The value of *k* is positively proportional to the size of the image [61]; therefore, we adopted *k* = 350 because the image size was 320 × 240 in this study.

Finally, the runoff components are identified under the guidance of the seed point:

(5) $$S_{water}=S^{m}∋v_{seed}\left(x,y\right)$$

In the formula, $v_{Seed}$ denotes the coordinates of the seed point in the image. The seed-containing component is identified as the runoff stream. Based on the above definitions and the graph-based segmentation method, we obtained the Algorithm 1.

**Algorithm 1** Seed-Guided Graph-Based SegmentationThe input is a graph G=(V, E), with n vertices and m edges. The output is a segmentation of V into components $S=\left(C_{1},\ldots ,C_{r}\right)$.
0.Sort E into $\pi \;=\;\left(o_{1},...,o_{m}\right)$ by non-decreasing edge weight.1.Start with a segmentation $S^{0}$, where each vertex$\;v_{i}$ is in its own component.2.Repeat step 3 for q = 1,...,m.3.Construct$\;S^{q}$ given $S^{q-1}$ as follows. Let $v_{i}$ and $v_{j}$ denote the vertices connected by the q-th edge in the ordering, *i.e.*, $o_{q}=(v_{i},v_{j})$. If $v_{i}$ and $v_{j}$ are in disjoint components of $S^{q-1}$ and $w\left(o_{q}\right)$ is small compared to the internal difference of both these components, then merge the two components; otherwise, do nothing. More formally, let $C_{i}^{q-1}$be the component of $S^{q-1}$ containing $v_{i}$ and $C_{j}^{q-1}$, the component containing $v_{j}$. If $C_{i}^{q-1}\neq C_{j}^{q-1}$ and $w\left(o_{q}\right)\leq MInt(C_{i}^{q-1},C_{j}^{q-1})$, then $\;S^{q}$ is obtained from $S^{q-1}$ by merging $C_{i}^{q-1}$ and $C_{j}^{q-1}$. Otherwise, $\;S^{q}=S^{q-1}$.4.All components of the image are obtained, and $S\;=\;S^{m}$. The guiding flood region $S_{water}$ is obtained from $S^{m}$ when $S^{m}$ is intersected by $v_{seed}\left(x,y\right)$.5.Return the waterbody region, $S_{water}$.

### 2.3. Calculation of Run off Fluctuation

Based on the interleaving degree of the visually detected waterbody region and virtual markers, the actual field water level fluctuation is calculated to determine the flood risk and water level. The virtual markers representing the actual boundaries are pre-labeled in the image. The flood risk of the runoff can be assessed according to the quantity, proportion, and order of the virtual markers covered by the waterbody. The water level fluctuation can be expressed by the proportion of virtual markers covered by the waterbody as follows:

(6) $$Water\;Level\;Fluctuation=\frac{\sum^{\text{​}}\left(VMs\in S_{water}\right)}{\sum^{\text{​}}VMs}$$

In this formula, $\sum^{\text{​}}VMs$ is the total number of virtual markers and $\sum^{\text{​}}\left(VMs\in S_{water}\right)$ is the number of virtual markers covered by the waterbody. The virtual markers placed on the surface and top of the riverbank can be used to reflect the extent of water overflow; those placed on the water gauges can be applied to evaluate water level fluctuation by using images. An actual example is presented in the following section.

## 3. Experimental Section and Discussion

### 3.1. Monitoring Embankment Overflow

Image monitoring was applied to monitor embankment overflow in this real-life case. The yellow “+” markers denote the virtual markers set on the embankment surface. The monitored location was the embankment of the MeiNong-Creek, which was monitored from 6:00 a.m. to 12:30 p.m. on 29 August 2013. During this period, this area was in the early rain phase of the Kong-Rey typhoon, and an overflow from the embankment occurred once. In the monitoring images, a residential area is adjacent to the MeiNong-Creek with the embankments serving as the boundaries. Five points were marked from the bottom to the top of the embankment to gauge the approaching water toward the embankment crown. The blue “*” markers denote the site where the seed was situated, which could be any site on the riverbed or runoff’s path to be sensed. Owing to the Kong-Rey typhoon, 498 mm of precipitation occurred within 24 h. Because the elevation of the MeiNong-Creek’s altitude is lower than that of its branches, overflow occurred continuously after noon, causing serious flooding on the roads of the adjacent MeiNong City. The local buildings, including post offices, fire stations, train stations, and agriculture associations, were flooded and paralyzed. In the downtown area, the average depth of the flood water reached 60 cm, and in the most critical flooded regions, the depth was 2 m. It was not until 8:00 p.m. that the flood ebbed and the roads were again operational.

**Figure 5:**
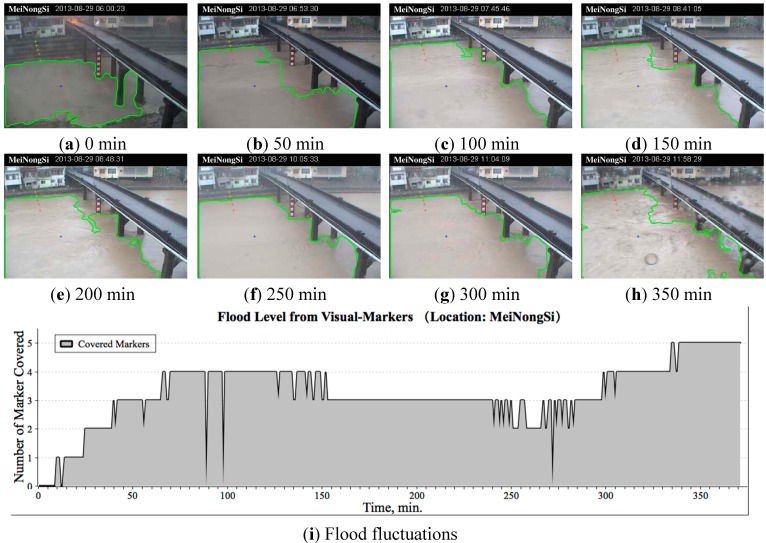
Real-time field images and analysis data of the flood level. In this case, only five virtual markers were set from the riverbed to the embankment top. (**a**–**h**) show the visual sensing results from screen shots every 50 min; and (**i**) the graph generated using the number of the virtual markers covered by the waterbody against time.

During this event, visual sensing was applied to acquire nearly real-time water level information, including analysis results of the runoff range from the monitoring images, extent of river water approaching the embankment, and time series fluctuation data. The visual analysis results and fluctuation data are shown in Figure 5. The monitoring images were sent back at a speed of one picture per minute; therefore, the sensing time resolution was 1 min. The analysis results with an interval of 50 min are shown in Figure 5a–h. The virtual markers are denoted with a yellow “+” and a red “+” if they are covered by water; the blue “*” denotes the seed. Figure 5i shows the visual analysis data of the river water fluctuation. The data are expressed as the number of flooded virtual markers on the embankment. The horizontal axis indicates the monitoring time from 0 min (starting time was 6:00 a.m.) to 370 min (12:30 a.m.); the vertical axis is the number of markers covered among the five markers in total. The number indicates the risk level; when all five markers were covered, a flood occurs. Figure 5i shows that high water levels occurred two times during the monitoring process. The first time, they appeared at around 8:00 a.m., when four markers were covered; the second time, they appeared at around 11:00 a.m., when all five markers were covered, indicating that overflow occurred continuously and led to a serious urban flood.

Visual sensing of monitoring images can clearly determine the water level fluctuation amplitude before the occurrence of overflow. Although the sensing is not based on the exact water level, it can be used for an initial automatic warning. For instance, if four flooded markers are defined as the warning threshold, an alert can be sent in advance to disaster prevention agencies to enable an early response to the subsequent flood event. In this way, these agencies can evacuate personnel and facilities, control access to flooded areas, and undertake protective actions of important institutions and key facilities prior to the occurrence of an urban flood.

**Figure 6:**
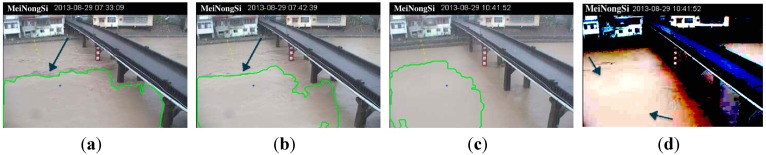
Images with false diagnoses. (**a**–**b**) boundaries are caused by wave of large turbulence, as indicated by the arrow; (**c**) result of signal loss that occurred during transmission and compression; and (**d**) result of contrast enhancement of (**c**) by 50 times.

Three false diagnoses were made during sensing in this actual case. Two false diagnoses occurred at the 89th and 98th minute, respectively, because the river was divided into upper and lower sections by a wave boundary on the water surface (Figure 6a–b). At the 272nd minute, there was significant signal loss in the image during the transmission and compression process from the remote site. During transmission, packet loss may typically be caused by network congestion between the remote site and the management center. During compression, lossy coding schemes are used to achieve the optimal image quality under bandwidth and power constraints, and this may degrade the perceptual image quality. This resulted in the incompleteness of segments owing to the obvious coding blocks (as shown in Figure 6c,d). Figure 6d shows a contrast enhancement of Figure 6c by 50 times.

### 3.2. Water Level Measurement

**Figure 7:**
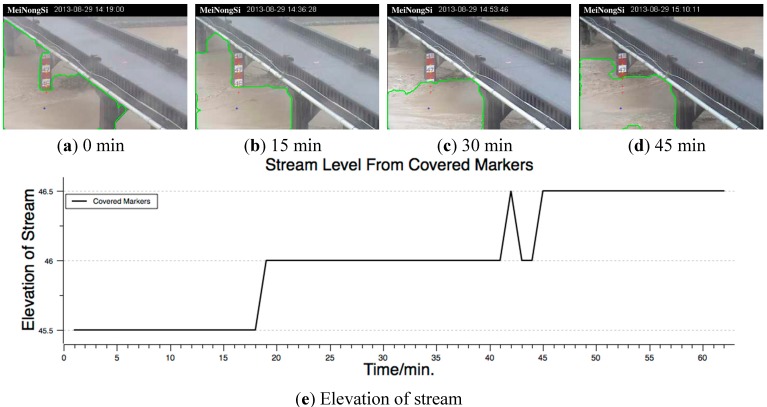
Indirect measurement of water level of runoff stream. The ruler scale in the images shows the elevation. (**a**–**d**) show the visual sensing results from screen shots every 15 min; and (**e**) the graph generated using the number of the virtual markers covered by the waterbody against time.

For the monitored site with a water ruler, the virtual markers can be set on the ruler’s scale so that the water level can be indirectly measured using images. In this example case, the monitored site was the ruler on the bridge pier on the MeiNong Creek, and the monitoring period was from 14:19 p.m. to 15:28 p.m. on 29 August 2013. During this period, the morning flood event had ended; however, several subsequent overflows aggravated the flood situation. The highest point of the water ruler was at an elevation of 48 m, and the top of the bank was at an elevation of ~46.5 m. When the water surface is above 46.5 m, it means that the water has overflowed the embankment. Virtual markers were set on the scale from 47.5 to 45.5 m at intervals of 0.5 m; in other words, the five markers respectively represented elevations of 47.5, 47, 46.5, 46, and 45.5 m. Figure 7 shows the monitoring results. Figure 7a–d show the results at intervals of 15 min, with virtual markers denoted by a yellow “+,” covered markers denoted by a red “+,” and a seed denoted by a blue “*.” Similar to the false diagnoses discussed in Section 3.1, fierce turbulence around the piers resulted in incomplete segmentation, as shown in Figure 7c.

At the 42nd minute (15:05 p.m.), the water level reached an elevation of 46.5 m, which was the same as the top of the bank on both sides; therefore, embankment overflow occurred, as shown in Figure 7e. Although water fluctuation caused the detected water level to decrease to 46 m at the 43rd and 44th minutes, subsequently, the actual water level was measured accurately. For disaster prevention and management, it is suggested that the water level of 46 m can be considered a warning level. Following this level, a warning was issued 25 min earlier in this example case. Moreover, increasing the number of markers can increase the precision of water level detection. However, for this purpose, the ruler scale area must be enlarged and the image quality should be improved. 

It is feasible to detect the water level by monitoring the juncture between the ruler and the waterbody using cameras. However, owing to the insufficient resolution of images (CIF is referenced in this paper), currently, the monitored FOV must be reduced to ensure the visibility of the ruler scale. For example, in this case, the ruler image must be enlarged two to four times to ensure the visibility of the water ruler. Although the detection precision was improved, the original purpose of monitoring the overall runoff condition was lost.

### 3.3. Monitoring of Specific Flood-Intruded Area

**Figure 8:**
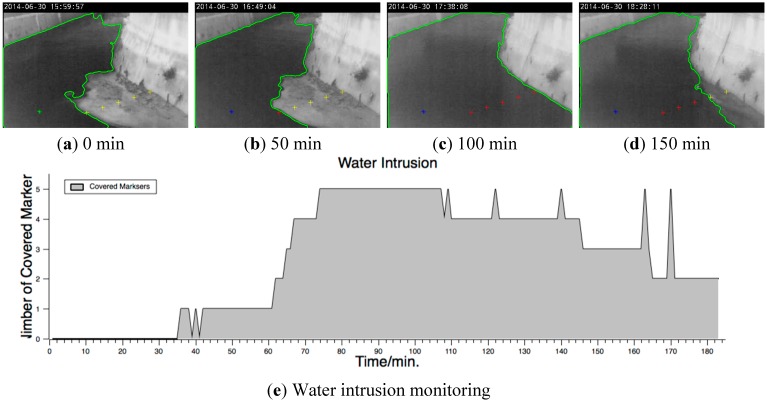
Water intrusion on specific ground measured using IR photography. (**a**–**d**) show the visual sensing results from screen shots every 50 min; and (**e**) the graph generated using the number of the virtual markers covered by the waterbody against time.

When no ruler or embankment in the monitored scenes can be used as a reference, or when the water intrusion of an actual space must be monitored, the virtual markers can be used to represent the physical boundaries of the image space. Figure 8 shows a case in which the monitored water intrusion caused ground flooding. In this case, the images were captured by infrared (IR) photography. The purpose of monitoring was to assess the flood severity of the wetland in the river shoal. Virtual markers were set on the river shoal from the edge of the stream to the embankment. A yellow “+,” red “+,” and blue “*” denote the virtual markers, covered markers, and seed, respectively. Figure 8a–d show the sensing results at intervals of 50 min, from which the change in flood severity with water level fluctuations at night can be identified clearly. Figure 8 shows the change in the water intrusion on the ground over time. The monitored place was completely flooded between the 72nd and 110th minutes. The flood began to ebb after the 145th minute.

IR imaging uses either added infrared light to form images via reflection over the object’s surface or thermal-radiation-derived scattered infrared light from the object itself to form images. Therefore, the images generated will not be influenced by insufficient light in the environment. Moreover, owing to a longer wavelength than that of visible light, IR light can penetrate heavy fog or rain to form images. Therefore, it is often used in satellite and aerial photographing to penetrate clouds [62,63]. At present, commercial surveillance IR photography primarily employs added IR light. Its FOV size is limited by the transmission and reflection ratios of IR light; consequently, this technology has yet to be widely applied to water resource management. Furthermore, IR technology transforms the actual scene to the surface light reflection intensity expressed as a grey-scale value; this results in a loss of color-induced visual features of objects. 

For example, in Figure 8e, the incorrect determinations at the 162nd and 169th minutes were caused by the weakened visual difference between the ground and the water surface owing to weak contrast in the IR images. IR imaging is the best way for monitoring in the evening or under inclement weather conditions. Visual sensing combined with the use of IR cameras can be applied for monitoring during low-light nighttime and under heavy rain or fog. However, because image processing is mostly developed for visual light emission images, the application of IR imaging in visual sensing should be explored further.

## 4. Conclusions

This study established an image-based visual sensing and water level analysis method. Through the automatic screening of images that contain visible content and the texture verification of a waterbody surface, abnormal water level fluctuations can be detected to enable the automatic sensing and early warning of flood events. Currently, early warnings for floods mainly rely on data from *in situ* measurements and remote sensing images. Field visual data of runoffs are not collectively applied in an effective manner. Consequently, dynamic field information required for timely decision-making to reduce disasters in small urban areas is insufficient. In this study, we therefore applied an image-based visual sensing method that uses passive monitoring cameras as visual sensors or “smart” cameras to automatically monitor flood fluctuations in a small urban area. The experiment results of this real flood case proved that the proposed method can accurately provide monitoring images and time series water level fluctuation data that reflects the current flood situation. Using this method, disaster prevention and management sectors can quickly and accurately understand the local hydrological conditions and efficiently implement disaster reduction measures.

Early flood prevention and water management techniques were predominantly based on hydrological engineering, such as embankments and containments. However, owing to the extreme climate changes in the last decade, resource investments in these projects have not produced satisfactory outcomes. Thus, in addition to hydrological engineering, new concepts such as risk avoidance and disaster reduction of floods have been introduced to flood management and control in recent years [64,65]. Using a relatively small investment, the current flood forecasting system aims to send early alerts for disaster prevention to control sectors so that disaster reduction actions can be implemented in a timely manner. These actions include opening a reservoir in advance to discharge floods, closing a water gate, and advance evacuation of people in low-lying areas. Existing warning systems mainly rely on monitoring stations for water level and precipitation information, remote sensing images, and numerical prediction systems. These systems can provide warnings of large-scale floods several hours or even 24 h in advance, and therefore, they remain the most widely used methods [10,23,66,67]. However, large-scale forecasting is not suitable for hazard reduction in small urban regions on account of its low specificity and timeliness. It is difficult to cross-verify the forecasting results and actual flood conditions. Thus, this system cannot provide sufficient information for decision-makers to manage disasters and to accurately and rapidly determine disaster reduction measures. In this paper, we therefore proposed an intelligent flood monitoring system using existing river monitoring cameras that can provide real-time field images and information regarding water level changes in a time series. By using this system, decision-makers can better understand the field situation to take appropriate actions for evacuations before the flood disaster occurs. Furthermore, they can apply suitable disaster reduction approaches afterwards, thereby reducing the flood’s impact on urban areas.

The monitoring of urban floods with VSNs can reduce field inspections by human beings while increasing the number of monitored sites. However, both VSNs and wireless VSNs (WVSNs) are highly reliant on the availability of electric power and communications. Therefore, when electric power and communications are interrupted by disasters, the continuity of monitoring becomes a significant bottleneck for sensing networks [68,69]. The measurement components of *in situ* measurement stations must be in contact with or stay on top of the water body, depending on the measurement method. Therefore, *in situ* sensors cannot survive well in the midst of the destruction caused by large-scale disasters. The biggest difference between VSNs and measurement stations is that VSNs are used to observe the overall environment instead of the water level. Therefore, image sensors can be placed at a greater distance in safety to ensure sufficient FOV and to have a higher chance of withstanding floods. If all measurement stations are damaged, the remote sensing images will be the only information source. Under this circumstance, the availability, reliability, and accuracy of data can only be guaranteed with the help of cyber physical systems (CPSs) that combine various monitoring and pre-warning systems, including site measurement instruments, remote sensing images, flood forecasting, and visual sensing [70,71]. 

In addition, the image segmentation method used in this study can operate very efficiently with a processing time of approximately 2 s for each CIF image, whereas the update interval of monitoring images is 1 min. Because the processing time is much shorter than the update time, the processing is nearly real-time without a calculation delay induced by data accumulation. However, in the future, with the increasing number of monitors and smart cameras deployed in urban areas and the growing need for high-resolution images, the real-time response time of virtual sensor systems will become a bottleneck; thus, more efficient algorithms [60,72] and distributed processes [69,73] must be considered.

Each flood monitoring and forecast system affords unique advantages. A single pre-warning system can accurately monitor and forecast floods under certain environmental conditions; however, it lacks an intelligent triggering and response function for field flooding and overflow events. Large-scale flood forecast systems cannot provide sufficient field information to decision-making agencies; thus, these agencies can obtain relevant data only from the vulnerable location. Therefore, there is a lack of flexibility in integrating integrate disaster prevention information and performing collective mitigation actions. Thus, an important future direction is to integrate various systems to determine appropriate disaster reduction measures. Managers require clear and specific disaster information to develop long-term management strategies and real-time disaster reduction actions. Toward this end, a key future research direction will be determining how to integrate field measurements, satellite sensing, and visual sensing into a practical information system, such as CPSs.
